# Forging a global shield against HMPV: Uniting healthcare systems and community action to counter a silent respiratory epidemic

**DOI:** 10.3934/publichealth.2026010

**Published:** 2026-01-21

**Authors:** Mona Gamal Mohamed, Shaimaa Hashem Elsalous

**Affiliations:** 1 Adult Health Nursing Department, RAK College of Nursing, RAK Medical and Health Sciences University, Ras Al Khaimah, United Arab Emirates; 2 Maternity and Newborn Health Nursing Department, RAK College of Nursing, RAK Medical and Health Sciences University, Ras Al Khaimah, United Arab Emirates

**Keywords:** human metapneumovirus, respiratory infections, pediatric pneumonia, viral diagnostics, vaccine development

## Abstract

**Background:**

Human metapneumovirus (HMPV) has emerged as a significant global cause of acute respiratory infections, disproportionately affecting vulnerable populations such as young children, the elderly, and immunocompromised individuals. Despite its considerable impact, contributing to 5%–15% of pediatric respiratory hospitalizations and 10% of respiratory deaths in children under five, HMPV often remains underdiagnosed and receives insufficient attention within public health frameworks. The absence of specific antiviral treatments and preventative vaccines further exacerbates its clinical consequences.

**Aim:**

This review aims to consolidate current understanding regarding HMPV's epidemiology, clinical manifestations, diagnostic approaches, and management strategies, while also identifying existing gaps in prevention and research efforts.

**Methods:**

A systematic review of peer-reviewed literature published between 2001 and 2024 was conducted. The search encompassed databases such as PubMed, Web of Science, and those maintained by the World Health Organization (WHO). Keywords utilized for the search included “human metapneumovirus”, “HMPV epidemiology”, and “HMPV treatment”. Studies selected for inclusion were rigorously assessed for their epidemiological data, diagnostic methodologies, clinical management protocols, and preventative measures. The collected data were then synthesized to pinpoint critical knowledge deficiencies and areas requiring future research focus.

**Results:**

Our comprehensive analysis indicates that HMPV contributes significantly to global morbidity, exhibiting seasonal patterns that vary geographically with hemisphere and climate. Diagnostic challenges persist due to the symptomatic overlap with other common respiratory viruses like RSV and influenza, and current treatment approaches remain primarily supportive. Promising advancements in vaccine development, including candidates targeting the F-protein and utilizing mRNA technology, are underway but necessitate further clinical evaluation. Populations at high risk, particularly those in resource-limited settings, experience a disproportionately higher burden of disease due to inadequate diagnostic capabilities and underdeveloped healthcare infrastructure.

**Conclusions:**

HMPV represents a substantial, yet frequently underestimated, respiratory pathogen demanding immediate public health attention. Key priorities for addressing this include (1) the development of rapid and accessible diagnostic tools, (2) accelerating vaccine candidates through rigorous clinical trials, (3) establishing evidence-based treatment guidelines, and (4) strengthening global surveillance systems. A coordinated international effort is imperative to mitigate the considerable health and economic repercussions of HMPV infection.

## 1. Introduction

Human metapneumovirus (HMPV) stands as a prominent, yet frequently overlooked, causative agent of acute respiratory infections (ARIs) globally. Its impact is particularly pronounced in pediatric populations, the elderly, and individuals with compromised immune systems. Despite its significant contribution to disease burden, accounting for approximately 5%–15% of pediatric ARIs and 10% of respiratory fatalities in children under five, HMPV's clinical presentation often mimics other viral infections, such as respiratory syncytial virus (RSV) and influenza, leading to diagnostic challenges that obscure its true prevalence [1]. Currently, there are no licensed antiviral medications or vaccines specifically targeting HMPV, meaning that patient management remains largely supportive. Furthermore, the emergence of distinct subtypes, such as lineage B, has been linked to an elevated risk of severe disease [2]. This review aims to comprehensively synthesize the existing knowledge concerning HMPV's epidemiology, pathogenic mechanisms, and global health implications. It will also critically evaluate current limitations in diagnostic and therapeutic approaches, while highlighting crucial research gaps to inform future public health strategies.

## 2. Methodology

### 2.1. Literature search and study selection

A systematic literature review was conducted on peer-reviewed articles published between 2001 and 2024, utilizing major databases such as PubMed, Web of Science, Scopus, and official World Health Organization (WHO) reports. The search strategy incorporated combinations of keywords including “human metapneumovirus”, “HMPV epidemiology”, “HMPV diagnostics”, and “HMPV treatment”. Boolean operators (AND/OR) were applied to refine search results, ensuring comprehensive coverage of both clinical and community-level studies related to HMPV.

### 2.2. Inclusion and exclusion criteria

Studies were included if they met the following criteria:

1. Original research or review articles with molecular or laboratory confirmation of HMPV infection.
2. Studies reporting quantitative or qualitative data on prevalence, clinical outcomes, or diagnostic modalities.
3. Sample sizes sufficient to allow meaningful interpretation of disease burden or management outcomes.

Exclusion criteria included:

1. Case reports, commentaries, or opinion pieces lacking primary data.
2. Articles not published in English.
3. Research with incomplete clinical or epidemiological information.

### 2.3. Data extraction and thematic analysis

Data extraction was performed independently by two authors using a standardized data collection template. Extracted data were categorized under three thematic domains:

1. Epidemiology and disease burden: prevalence trends, high-risk populations, and mortality rates.
2. Clinical management: diagnostic strategies, therapeutic interventions, and associated complications.
3. Policy and preparedness gaps: global surveillance mechanisms, vaccine development, and coordinated public health responses.

Discrepancies in extracted data were reconciled through discussion to ensure consistency and accuracy.

### 2.4. Quality assessment and risk of bias evaluation

In response to reviewer recommendations, a detailed quality assessment was incorporated. Each included study underwent a formal risk-of-bias evaluation using the Newcastle–Ottawa Scale (NOS) for observational studies and the Cochrane Risk of Bias tool for randomized controlled trials. The assessment focused on selection bias, comparability, and outcome reporting. Studies were categorized as low, moderate, or high risk of bias based on consensus between reviewers. Disagreements were resolved through consultation with a third reviewer to ensure objectivity and methodological rigor.

### 2.5. PRISMA flow diagram

**Figure 1.**
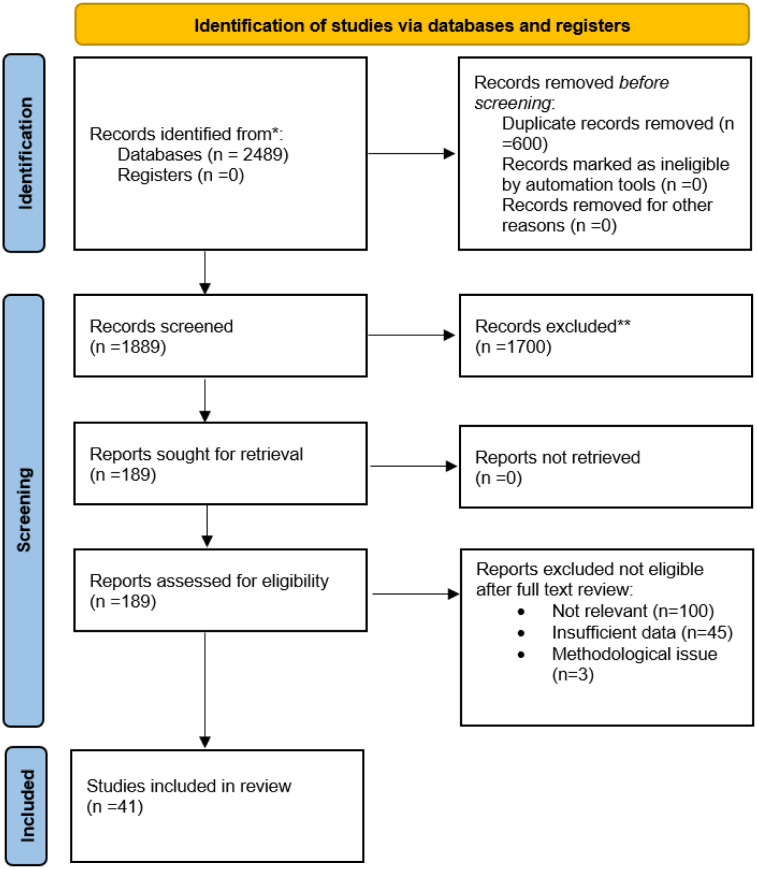
PRISMA flow diagram.

A PRISMA flow diagram (Figure 1) was added to illustrate the study selection process, detailing the total number of records identified, screened, excluded, and finally included in the review. This addition enhances transparency, replicability, and methodological credibility of the review process.

## 3. Epidemiology and global burden of HMPV

Respiratory infections are globally pervasive, with children disproportionately affected [3]. In developed nations, these infections are a primary cause of hospitalization and mortality among children under five [4]. For instance, in Brazil, respiratory infections contribute to 22.3% of annual deaths in children aged 1–4 years [5]. Human metapneumovirus (HMPV) is a pervasive cause of acute respiratory infections (ARIs) worldwide, with nearly all children exposed by age five [6],[7]. Detection rates in pediatric cohorts range from 5% to 15%, contributing to 5%–10% of hospitalizations due to ARIs [8]. Retrospective analyses indicate that HMPV circulated undetected for decades before its first isolation in 2001 [9],[10]. Seasonal trends vary by geography: in temperate regions, incidence peaks during late winter and early spring, often overlapping with RSV and influenza surges, while tropical regions may experience higher activity during rainy seasons [11],[12]. Environmental factors, such as humidity and rainfall, also modulate viral transmission. HMPV's burden is particularly significant in infants, young children, the elderly, and immunocompromised individuals, highlighting the global public health impact of this “silent” pathogen [13].

## 4. Clinical presentation, complications, and high-risk populations

HMPV infection presents along a spectrum from mild, cold-like symptoms to severe lower respiratory tract involvement. Children, especially those under two, often develop fever, cough, wheezing, bronchiolitis, or pneumonia. Infants may exhibit poor feeding, irritability, or apnea, while adults usually experience mild influenza-like symptoms [14],[15]. High-risk populations, including the elderly, immunocompromised patients, pregnant women, and those with chronic conditions such as asthma or COPD, are more susceptible to severe disease complications (e.g., ARDS, secondary bacterial infections) and prolonged recovery [16],[17]. Symptom onset typically occurs 3–6 days post-exposure, with mild cases resolving within 1–2 weeks; severe cases may require hospitalization, oxygen therapy, or mechanical ventilation [18],[19] (Table 1). Management remains supportive, emphasizing hydration, rest, symptomatic treatment, and preventive hygiene measures, while ongoing research explores vaccine development and targeted antivirals [20].

**Table 1.** Spectrum of clinical presentation and severe outcomes by population group.

Population	Common symptoms	Severe manifestations
Children (<5 y)	Fever, cough, wheezing	Bronchiolitis, pneumonia
Infants (<2 y)	Poor feeding, irritability, apnea	ICU admission (3/1000 infants)
Adults	Nasal congestion, sore throat, low-grade fever	Pneumonia, COPD exacerbation
Elderly/immunocompromised	Fatigue, myalgia	ARDS, secondary bacterial infections

## 5. Mechanisms of HMPV infection and immune response in pediatric patients

The symptomatology observed in HMPV infections is primarily driven by a characteristic Th17-like immune response, marked by the pulmonary secretion of interleukin (IL)-6 and tumor necrosis factor-alpha (TNF-α). This immune reaction is often accompanied by an insufficient Th2-like profile, evidenced by the early release of pro-inflammatory cytokines such as IL-4, IL-5, and IL-8 [21]. Notably, thymic stromal lymphopoietin (TSLP), a cytokine known to impede T-cell activation, contributes to a delayed Th1-like immunity and promotes the secretion of Th2-related cytokines. This leads to significant neutrophil infiltration in the lungs of infected mice [21]–[24]. This aberrant immune response, coupled with excessive mucus production by goblet cells, can result in the collapse of respiratory airways [25]. HMPV infection in children under two years of age is recognized as a risk factor for the subsequent development of asthma, a phenomenon also observed with human respiratory syncytial virus (HRSV). Furthermore, in some severe cases, HMPV has been implicated in the progression of chronic obstructive pulmonary disease (COPD) and exacerbated asthmatic responses in humans [26]. It is estimated that HMPV is associated with approximately 10%–12% of respiratory illnesses in children, making it one of the most prevalent viruses leading to hospitalization in young children [27]. Studies have also indicated that HMPV contributes to hospitalization rates of 1 in 1000 children under five years and 3 in 1000 infants under six months. Moreover, the prevalence of HMPV infection has been reported to be comparable to that of the influenza virus and parainfluenza virus types 1–3 [28].

**Figure 2.**
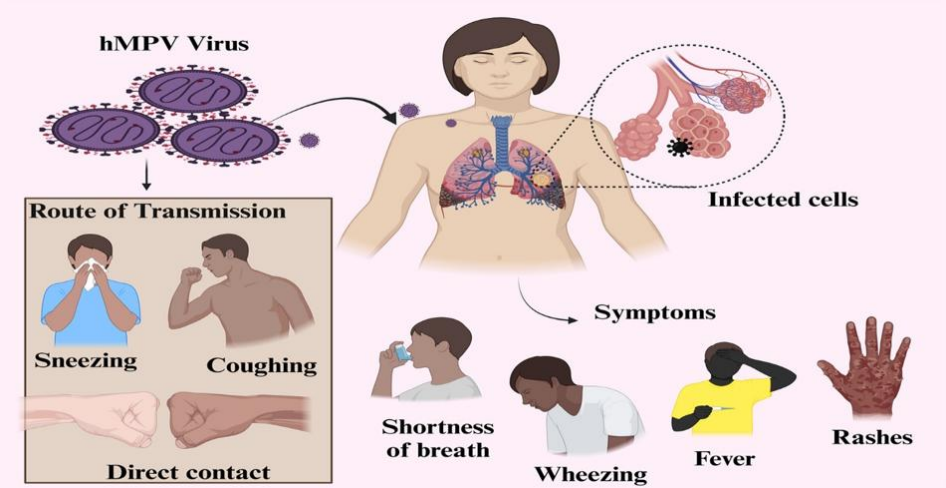
Pathogenesis, transmission, and clinical manifestations of human metapneumovirus (HMPV) infection.

HMPV accounts for approximately 15% of acute respiratory infections in young children and about 5% in adults [29]. According to a 2017 World Health Organization report, HMPV was responsible for 10% of deaths in children under five years of age. In 2018, it was estimated that 14 million children under five worldwide were infected with HMPV, leading to 640,000 hospitalizations and 8000 deaths [30]. While HMPV infection typically results in mild symptoms such as fever, cough, and nasal congestion, individuals with compromised immune function, such as premature infants, may develop severe complications, including bronchiolitis, pneumonia, and even severe pneumonia requiring intensive care unit (ICU) admission. Overall, HMPV imposes a substantial annual medical burden, particularly in middle- and low-income countries. Therefore, targeted intervention strategies are critically needed to protect infants and young children [31] (Figure 2).

## 6. Diagnostic challenges of human metapneumovirus

Accurate diagnosis of HMPV remains challenging due to its clinical and radiological overlap with other respiratory pathogens. Current diagnostic methods primarily include molecular techniques such as polymerase chain reaction (PCR), which provide high sensitivity and specificity for detecting HMPV RNA in respiratory specimens [32]. Despite their accuracy, PCR assays are costly, technically complex, and require specialized laboratory infrastructure, limiting their availability in low-resource settings [33]. Rapid antigen detection tests, while more accessible and faster, demonstrate reduced sensitivity, particularly in early-stage or mild infections, contributing to false-negative results and underdiagnosis [34]. Serological assays can identify HMPV-specific antibodies but are limited to retrospective diagnosis because antibody responses appear only weeks after infection, offering minimal utility for acute management [35].

The nonspecific clinical presentation of HMPV, including fever, cough, and wheezing, further complicates differential diagnosis, as these symptoms are shared with influenza, RSV, and rhinovirus infections [35]. Radiological findings, such as infiltrates or consolidation, are similarly non-distinctive, necessitating the integration of molecular, clinical, radiological, and epidemiological data for accurate identification [36]. Even high-quality diagnostics may yield false negatives if viral load is low or sample collection is suboptimal, underscoring the need for standardized testing protocols.

Surveillance gaps exacerbate these diagnostic challenges. Many health systems do not routinely test for HMPV, resulting in underreporting and misclassification of cases under more common pathogens. Empirical treatment with antibiotics or corticosteroids can mask the true prevalence, hindering epidemiological tracking and targeted intervention planning [37]. Integration of HMPV diagnostics into existing influenza and RSV surveillance platforms, such as multiplex PCR panels routinely used in national sentinel sites, has shown promise in high-income countries, providing timely detection and informing outbreak response [38]. Low and middle-income countries could adapt these models by leveraging centralized laboratory networks or point-of-care molecular platforms, combined with digital reporting systems, to improve detection and reporting rates.

Addressing these gaps requires a multifaceted strategy: expanding access to molecular diagnostics, increasing clinician awareness, standardizing testing protocols, and integrating HMPV surveillance into broader respiratory virus monitoring programs. Strengthened diagnostic and surveillance capacity will enable accurate burden assessment, inform timely clinical management, and support evidence-based public health interventions.

## 7. Treatment and management strategies

Treatment for HMPV infection primarily involves supportive care, as there are currently no licensed antiviral medications specifically targeting the virus. Approaches such as immunoglobulins, glucocorticoids, and other symptomatic treatments are considered the mainstays of management. In clinical practice, ribavirin and polyclonal intravenous immune globulin (IVIG) have been investigated as potential treatments. Ribavirin, a nucleoside with activity against RNA viruses, has demonstrated in vitro activity against HMPV and showed some efficacy in mouse models [39]. Commercial IVIG preparations contain neutralizing activity against HMPV, and antibodies alone have exhibited prophylactic and therapeutic efficacy in mice. While these two treatment options are employed in severe infection cases, there is a notable lack of studies exploring their definitive effectiveness in human patients, and further randomized controlled trials (RCTs) are needed. Despite this, these therapies are still indicated based on clinical experience. Anecdotal reports exist regarding human use of ribavirin and IVIG, but controlled trials and formal guidelines for their recommendation are absent [40].

Immunomodulators have been explored as supplementary therapies for severe HMPV cases, particularly in immunocompromised individuals. Intravenous immunoglobulin (IVIG) has been administered in conjunction with antiviral agents like ribavirin to bolster the immune response against the virus. IVIG contains neutralizing antibodies that may potentially reduce viral load and inflammation. However, its use carries risks such as fluid overload and allergic reactions, especially in children with congenital heart disease [41].

Corticosteroids have also been considered for their anti-inflammatory properties, but their role in HMPV management remains contentious. While some studies suggest that corticosteroids might help reduce inflammation and improve outcomes in severe cases, others have found no significant benefit or even potential harm, such as increased viral replication or immunosuppression [42]. Further research is necessary to clarify the appropriate application of immunomodulators in HMPV treatment.

## 8. The role of vaccination: Current developments and future prospects

The development of an effective human metapneumovirus (HMPV) vaccine is fraught with significant challenges. A primary obstacle is the virus's inherent capacity to circumvent the host immune system. HMPV can propagate directly from cell to cell, even in the presence of neutralizing antibodies, which complicates the design of vaccines capable of effectively blocking transmission [43].

Furthermore, the immune response to HMPV is multifaceted, involving both antibody-mediated and T-cell-mediated immunity. While the fusion (F) protein is a key target for vaccine development due to its ability to elicit neutralizing antibodies, it has been observed that the levels of such antibodies can rapidly diminish over time. This limits the long-term efficacy of vaccines relying solely on the F protein. Additionally, pre-existing immunity from prior infections can influence the clinical outcomes of vaccination, making it difficult to formulate a vaccine that performs consistently across diverse age groups and immune statuses [44].

The feasibility of implementing widespread HMPV immunization programs hinges on several factors, including the disease burden, existing healthcare infrastructure, and public health priorities. HMPV is a substantial cause of respiratory infections, particularly impacting young children and the elderly. Given the high prevalence of the virus and the potential for vaccines to reduce morbidity and mortality, there is a compelling rationale for mass immunization [45]. However, the success of such initiatives would necessitate robust healthcare systems capable of efficient vaccine distribution, storage, and administration. Moreover, public awareness and acceptance of vaccination are paramount. Educational campaigns and community engagement efforts would be essential to ensure high vaccination coverage. Lessons learned from mass vaccination programs for other respiratory viruses, such as influenza and RSV, could serve as a valuable model for HMPV immunization. International support and collaborative efforts would also be advantageous in addressing the logistical and financial hurdles associated with large-scale vaccination endeavors [46].

## 9. Strengthening healthcare systems for HMPV preparedness

Establishing robust and comprehensive surveillance systems is paramount for the early detection and continuous monitoring of HMPV infections. These systems should encompass detailed data collection on HMPV cases, including not only basic demographic information but also specific clinical symptoms, disease progression, and patient outcomes [47]. Timely reporting mechanisms must be established to ensure that healthcare providers and public health authorities are promptly alerted about emerging outbreaks, facilitating a swift and coordinated response. Rapid diagnostic tests should be made widely available, particularly in primary care settings and emergency departments, to facilitate the early identification of HMPV cases. This includes not only the provision of testing kits but also the necessary infrastructure and trained personnel to handle the testing process efficiently [48]. Training healthcare professionals in the proper use and accurate interpretation of these tests is essential to ensure reliable and timely diagnostic results, which can significantly impact patient management and infection control measures.

HMPV should be formally and strategically integrated into national respiratory disease strategies to ensure a coordinated and effective response to this significant public health concern. This involves developing specific, evidence-based guidelines and protocols for the prevention, diagnosis, and treatment of HMPV infections. Public health campaigns should be launched to raise awareness about HMPV among both the general population and healthcare professionals [49]. These campaigns should emphasize the importance of vaccination (when available), proper hygiene practices, and the need for timely medical care. Collaboration with international health organizations can provide valuable resources, expertise, and funding to strengthen national strategies and enhance the capacity to respond to HMPV outbreaks effectively [50].

**Figure 3.**
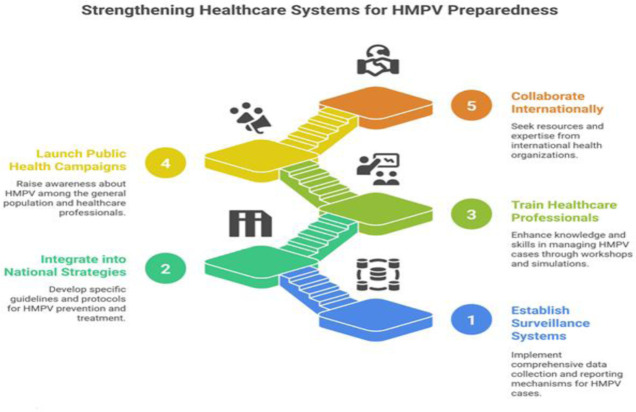
A strategic framework for healthcare system preparedness against HMPV.

Healthcare professionals play a pivotal role in the rapid response to HMPV outbreaks, and their preparedness is critical to mitigating the impact of the virus. Training programs should be implemented to enhance their knowledge and skills in managing HMPV cases. This includes organizing workshops and seminars that cover the latest diagnostic tools, treatment options, and infection control measures [51]. Simulation exercises can be invaluable in helping healthcare teams practice responding to HMPV outbreaks in a controlled environment, thereby improving their readiness and coordination during actual outbreaks. Continuous education through online platforms, professional journals, and conferences should be encouraged to keep healthcare professionals updated on the latest developments in HMPV research and management. Additionally, creating networks and forums for healthcare professionals to share experiences and best practices can further enhance their ability to respond effectively to HMPV challenges [52],[53] (Figure 3).

## 10. Community engagement and public health interventions for HMPV

Raising public awareness about HMPV through targeted educational campaigns is a foundational step in controlling its dissemination. These campaigns should be meticulously designed to reach diverse populations, including children, adults, the elderly, and individuals with underlying health conditions. The content must be adapted to various age groups and cultural contexts, ensuring accessibility and relevance for all [54]. Key messages should underscore the importance of recognizing HMPV symptoms, understanding transmission pathways, and knowing when to seek medical attention. Public health agencies can leverage multiple channels, such as social media, community workshops, school-based programs, and local media outlets, to disseminate this vital information [55]. Collaborations with community leaders, healthcare providers, and educators can significantly amplify the reach and effectiveness of these campaigns, fostering a collective sense of responsibility in preventing HMPV infections [56].

Enhancing hygiene and infection control measures is paramount to curtailing HMPV transmission within communities. Emphasizing the critical importance of regular handwashing with soap and water for at least 20 seconds is essential, as it stands as one of the most effective strategies to prevent the spread of respiratory viruses. In environments where handwashing facilities are limited, the promotion of alcohol-based hand sanitizers containing at least 60% alcohol is crucial. Encouraging individuals to cover coughs and sneezes with a tissue or the inner elbow, followed by proper tissue disposal and hand hygiene, can substantially reduce transmission risk [57]. Routine disinfection of frequently touched surfaces, including doorknobs, countertops, and electronic devices, should be standard practice in homes, schools, and workplaces. Furthermore, advocating for mask usage, particularly during peak HMPV seasons or in crowded settings, provides an additional layer of protection. Public health messaging should consistently reinforce these practices to ensure their integration into daily routines [58].

Schools, workplaces, and religious centers play pivotal roles in HMPV mitigation efforts, serving as key hubs for social interaction and potential viral transmission. Schools should implement stringent sick policies, encouraging students and staff exhibiting respiratory symptoms to remain home until they are no longer contagious. Providing accessible handwashing stations, sanitizers, and educational materials about HMPV can contribute to a healthier environment [59]. Workplaces can adopt flexible sick leave policies and promote remote work options during outbreaks to minimize exposure. Prioritizing regular cleaning and ventilation of shared spaces is also vital. Religious centers can contribute by encouraging adherence to hygiene practices during gatherings and providing hand sanitizer stations. Leaders within these settings can serve as role models by demonstrating proper hygiene behaviors and reinforcing public health messages. Through collaborative efforts, these institutions can significantly reduce the spread of HMPV and safeguard community health [60].

## 11. Policy and collaborative efforts around the globe

Regional and international partnerships are indispensable in the global endeavor to combat HMPV. These collaborations facilitate the crucial exchange of knowledge, resources, and expertise necessary to effectively manage the virus's spread. Organizations such as the World Health Organization (WHO) and the Centers for Disease Control and Prevention (CDC) are instrumental in coordinating efforts among nations and international health agencies [61]. They work closely with national health authorities to implement unified strategies for disease surveillance, early detection, and outbreak response. These partnerships also contribute to the development of standardized diagnostic protocols and treatment guidelines, ensuring a consistent approach to HMPV case management across diverse regions. Furthermore, regional alliances can address health disparities by supporting countries with limited healthcare infrastructure, providing technical assistance and financial aid to bolster their capacity to respond to HMPV outbreaks [62].

Governmental policies are critical for establishing and sustaining robust respiratory disease surveillance systems. These policies should mandate the systematic reporting of HMPV cases to public health authorities, enabling timely data collection, analysis, and interpretation. Governments must allocate resources to enhance laboratory capacity for HMPV testing, ensuring the availability of rapid diagnostic tools in healthcare settings [63]. This includes investing in advanced molecular diagnostic equipment and training laboratory personnel to accurately handle and interpret test results. Moreover, policies should promote the integration of HMPV into national respiratory disease strategies, emphasizing prevention, early detection, and effective outbreak management. Public health campaigns, supported by government policies, can raise awareness about HMPV and encourage adherence to preventive measures among the population. These campaigns should be tailored to different demographic groups and cultural contexts to maximize their reach and impact [64].

## 12. Future directions and research gaps in human metapneumovirus (HMPV) research

The development of effective therapeutics and vaccines for HMPV remains a critical area of ongoing research. Recent advancements in AI-guided vaccine design have demonstrated considerable promise, particularly in the creation of a double-cleaved, stabilized, and closed HMPV Pre-F trimer [65]. This innovative approach has shown robust protective efficacy in animal models, offering a potential pathway for future vaccine development. Additionally, research into virus-like particle (VLP) vaccines has indicated potential for protection against HMPV infections, with studies revealing effective immune responses in preclinical models. The application of mRNA vaccine technology, which has proven successful against other respiratory viruses, is also being explored for HMPV, with preliminary results suggesting strong immune responses [66]. However, further extensive research is imperative to optimize these technologies and rigorously confirm their safety and efficacy in human populations.

Comprehensive epidemiological data on HMPV are essential for a thorough understanding of its global burden and for the formulation of effective public health strategies. Current data underscore the significant impact of HMPV on respiratory health, especially within vulnerable populations such as children and the elderly. Nevertheless, substantial gaps persist in our understanding of the virus's transmission dynamics, seasonal patterns, and associated risk factors. Enhanced surveillance systems and standardized reporting mechanisms are necessary to collect accurate and timely data on HMPV infections. This data should then be utilized to inform public health policies and guide the allocation of resources for prevention and control efforts [67]. Furthermore, research into the long-term outcomes of HMPV infections and their economic impact will aid in prioritizing HMPV within the broader landscape of public health challenges.

Cross-border collaboration is vital for the effective control of HMPV, given the virus's capacity for rapid international spread. Lessons gleaned from previous cross-border collaborations on other infectious diseases highlight the importance of coordinated efforts in surveillance, prevention, and response. Initiatives such as the Mekong Basin Disease Surveillance (MBDS) and the Bangladesh, Bhutan, India, and Nepal (BBIN) Project exemplify the value of collaborative mechanisms in maximizing disease control activities [68]. However, these efforts frequently encounter challenges related to sustainability and follow-up actions. To strengthen cross-border collaboration, regular meetings, joint strategic planning, and pilot projects should be established to address shared health threats. International organizations, such as the WHO, play a crucial role in facilitating these collaborations and providing guidance on implementing effective cross-border health management strategies. By fostering partnerships and sharing resources, countries can collectively enhance their ability to detect, respond to, and control HMPV outbreaks [69]. The development of effective vaccines remains a paramount objective in HMPV research. Live attenuated and subunit vaccines show promise due to their capacity to stimulate robust immune responses with potentially fewer side effects. Novel delivery platforms, such as nanoparticles and viral vectors, could revolutionize vaccine administration and efficacy. The ongoing search for new antiviral agents continues, with researchers investigating compounds capable of effectively inhibiting viral replication while minimizing host cell toxicity [70].

Immunomodulatory approaches are gaining increasing attention as our understanding of the delicate balance between protective immunity and immunopathology becomes clearer. Scientists are actively exploring combination therapies, recognizing that targeting multiple facets of viral infection and host response may lead to superior clinical outcomes. Diagnostic capabilities require substantial enhancement to improve patient care. The development of rapid point-of-care tests would enable swift clinical decision-making, potentially reducing disease transmission and improving treatment outcomes. Identifying reliable biomarkers that correlate with disease severity and progression remains a critical research priority. Such markers could assist clinicians in predicting which patients may require more intensive intervention. Additionally, creating robust severity prediction tools would facilitate more efficient resource allocation within healthcare settings [71].

**Figure 4.**
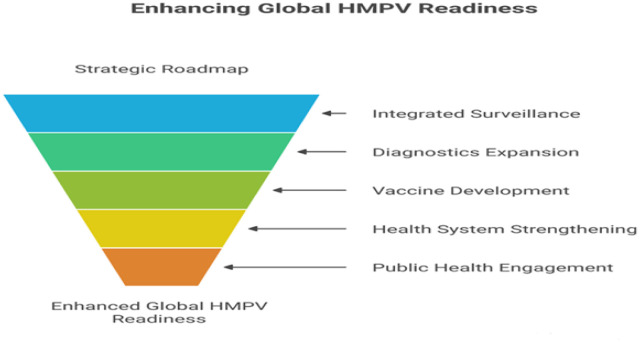
Strategic framework for enhancing global HMPV preparedness.

Strengthening health systems is fundamental for effective HMPV management. This entails leveraging current responses to enhance both pandemic preparedness and routine health services, investing in essential public health functions, including all-hazards emergency risk management, and building a strong primary healthcare foundation. Institutionalized mechanisms for whole-of-society engagement should be established to ensure coordinated efforts across various sectors. Creating and promoting enabling environments for research, innovation, and learning is crucial, as is increasing domestic and global investment in health system foundations and emergency risk management [72]. Addressing pre-existing inequities and the disproportionate impact of diseases on marginalized and vulnerable populations is also vital. Effective interdisciplinary and cross-sector collaborations significantly enhance the capacity to respond to health emergencies. Clear and streamlined governance structures are essential for coordinated efforts across various entities, facilitating swift decision-making and resource allocation. The robustness of pre-existing research infrastructures plays a crucial role in the rapid mobilization of resources and execution of large-scale research projects. Knowledge mobilization efforts are vital in disseminating research findings promptly to inform public health responses. Continuous tracking and evaluation of health research activities enable real-time adjustments and informed decision-making. Rapid identification and funding of research priorities, including vaccine and therapeutic development, are critical in addressing urgent public health needs. Effective resource allocation and capacity-building efforts ensure focused and accelerated research responses. Comprehensive strategic planning, involving stakeholder engagement and robust monitoring tools, is essential for aligning research efforts with health system needs [73] (Figure 4).

## 13. Conclusions

Human metapneumovirus (HMPV) remains a significant and frequently under-recognized respiratory pathogen, contributing substantially to morbidity and mortality, particularly among children under five, the elderly, and immunocompromised populations. Epidemiological data indicate that HMPV accounts for 5%–15% of pediatric acute respiratory infections (ARIs) and up to 10% of respiratory-related deaths in children under five, highlighting its global health impact. Despite this burden, underdiagnosis persists due to clinical overlap with RSV and influenza, limited access to molecular diagnostics in resource-constrained settings, and suboptimal integration of HMPV into routine surveillance systems. Management continues to rely primarily on supportive care, leaving high-risk populations vulnerable to severe complications such as bronchiolitis, pneumonia, and acute respiratory distress syndrome (ARDS).

HMPV's seasonal peaks, genetic diversity (subtypes A and B), and ability to evade host immunity further complicate prevention and treatment strategies. Current literature underscores substantial gaps in vaccine development, diagnostic coverage, and global surveillance infrastructure. To address these challenges, we propose a framework for global HMPV readiness, emphasizing six pillars: integrated surveillance and monitoring, expanded diagnostics, accelerated vaccine development, health system strengthening, public health and community engagement, and targeted research on epidemiology and immunopathogenesis. Implementing this framework can guide policymakers, clinicians, and researchers toward coordinated strategies that enhance detection, optimize clinical management, and reduce the global burden of HMPV.

## 14. Limitations

Despite efforts to conduct a comprehensive and systematic review, several limitations should be acknowledged. First, the search was limited to publications in English, which may have excluded relevant studies in other languages. Second, heterogeneity in study designs, sample sizes, diagnostic methods, and reporting standards across included studies may limit direct comparability and generalizability of findings.

## 15. Recommendations and future directions

### 15.1. Strengthen global surveillance

Our review found limited epidemiological data from low- and middle-income countries, creating blind spots in HMPV detection and outbreak response. Implementing standardized case definitions and integrated reporting systems can generate reliable incidence and prevalence data, facilitating a timely detection of outbreaks.

### 15.2. Accelerate vaccine development

Evidence from vaccine studies, including F-protein and mRNA-based candidates, demonstrates potential immunogenicity, yet no vaccine is currently approved. Targeting high-risk populations identified in our review—infants, the elderly, and immunocompromised individuals—should be a priority, leveraging insights from RSV and SARS-CoV-2 vaccine platforms.

### 15.3. Enhance diagnostic capabilities

Multiplex PCR assays, though effective, remain underutilized, particularly in resource-limited settings. Our findings indicate that expanding access to rapid, accurate diagnostics will improve case recognition and guide appropriate management.

### 15.4. Promote longitudinal and multicenter research

Data gaps on reinfection rates, seasonal patterns, and co-infection dynamics highlight the need for large-scale, longitudinal studies. Understanding host immune responses and genetic susceptibility could inform novel therapeutic targets and preventive strategies.

### 15.5. Assess health system impact

The literature indicates substantial hospitalization rates, ICU admissions, and healthcare resource utilization due to HMPV, especially in pediatric and high-risk adult populations. Economic evaluations and resource-use assessments can inform policy prioritization and resource allocation.

### 15.6. Public education and prevention campaigns

Awareness initiatives emphasizing hygiene practices and HMPV's clinical significance are critical for infection control and can reduce transmission in community and healthcare settings.

Collectively, these data-driven measures address identified knowledge gaps and provide a roadmap for developing targeted interventions to reduce the global burden of HMPV.

## Use of AI tools declaration

The authors declare they have not used Artificial Intelligence (AI) tools in the creation of this article.
